# Use of machine learning techniques for identifying ischemic stroke instead of the rule-based methods: a nationwide population-based study

**DOI:** 10.1186/s40001-023-01594-6

**Published:** 2024-01-03

**Authors:** Hyunsun Lim, Youngmin Park, Jung Hwa Hong, Ki-Bong Yoo, Kwon-Duk Seo

**Affiliations:** 1 Department of Research and Analysis, National Health Insurance Service Ilsan Hospital, Goyang, Republic of Korea; 2 Department of Family Medicine, National Health Insurance Service Ilsan Hospital, Goyang, Republic of Korea; 3 Division of Health Administration, Yonsei University, Wonju, Republic of Korea; 4 Department of Neurology, National Health Insurance Service Ilsan Hospital, Goyang, Republic of Korea; 5 Department of Neurology, Graduate School of Medicine, Kangwon National University, Chuncheon, Republic of Korea

**Keywords:** Phenotyping, Ischemic stroke, Machine learning, Deep learning, Insurance claim analysis

## Abstract

**Background:**

Many studies have evaluated stroke using claims data; most of these studies have defined ischemic stroke using an operational definition following the rule-based method. Rule-based methods tend to overestimate the number of patients with ischemic stroke.

**Objectives:**

We aimed to identify an appropriate algorithm for identifying stroke by applying machine learning (ML) techniques to analyze the claims data.

**Methods:**

We obtained the data from the Korean National Health Insurance Service database, which is linked to the Ilsan Hospital database (*n* = 30,897). The performance of prediction models (extreme gradient boosting [XGBoost] or gated recurrent unit [GRU]) was evaluated using the area under the receiver operating characteristic curve (AUROC), the area under precision–recall curve (AUPRC), and calibration curve.

**Results:**

In total, 30,897 patients were enrolled in this study, 3145 of whom (10.18%) had ischemic stroke. XGBoost, a tree-based ML technique, had the AUROC was 94.46% and AUPRC was 92.80%. GRU showed the highest accuracy (99.81%), precision (99.92%) and recall (99.69%).

**Conclusions:**

We proposed recurrent neural network-based deep learning techniques to improve stroke phenotyping. This can be expected to produce rapid and more accurate results than the rule-based methods.

**Supplementary Information:**

The online version contains supplementary material available at 10.1186/s40001-023-01594-6.

## Introduction

Stroke is the second leading cause of death worldwide and often causes disabilities among survivors. The incidence and prevalence of stroke have been increasing over the past 30 years [1, 2] and vary depending on the population structure and country’s economic level; however, ischemic stroke accounts for 80% and hemorrhagic stroke accounts for 20% of all cases [2]. In high-income countries, the age-specific stroke incidence has dramatically decreased due to the provision of preventive treatment and implementation of lifestyle changes; however, the number of new stroke cases is expected to increase with the aging of the population [3]. Stroke also imposes a significant burden on healthcare systems due to the long-term costs associated with disability. Therefore, it is an important outcome variable or independent variable in medical research.

Stroke is a disease with high incidence and prevalence; as such, active researches are conducted using large data sets. Administrative data obtained from large data sets can accurately reflect the real-world practices, are population-based, and can be used as a basis for long-term follow-up evaluations [4]. However, administrative data are not originally intended for research and have several limitations, one of which is the suboptimal accuracy of the assigned International Classification of Disease (ICD)-10 diagnostic codes. The selection of diagnosis codes depends on the patient’s primary diagnosis and can be affected by the accuracy of the physician’s diagnosis [5]. Diagnosis coding can also be affected by the financial incentives provided to the corresponding hospitals [6]. To overcome these limitations, several studies have established operational definitions of stroke diagnosed a by neurologist and have validated them [7]. Relevant studies have also been conducted to construct algorithms for identifying ischemic stroke using claims data by linking the patient’s information obtained from multicenter registries to the claims data and verifying the key identifiers [8].

Machine learning (ML) is an analytical method that uses computerized algorithms to identify the relationships among large amounts of data and to make predictions. In stroke-related studies, ML techniques are used to identify and classify strokes, predict stroke outcomes, and identify the stroke subtypes. It helps researchers to analyze large amounts of data, identify patterns, and make predictions that can be useful for the diagnosis, treatment, and prognosis prediction of stroke patients [9]. ML techniques are used to analyze the data obtained from the electronic health records (EHRs) to differentiate ischemic stroke from hemorrhagic stroke. Studies have been conducted to determine the extent to which these techniques can accurately detect the appropriate patients confirmed by experts [10]. A previous study used ML techniques to analyze EHR data in order to identify patients with acute ischemic stroke [11]. Although several previous studies have developed ML algorithms for identifying stroke using EHR data, it remains uncertain whether these algorithms can produce the same results in other countries with different healthcare systems. The performance of such algorithms may be affected by various factors such as the availability and quality of data, specific healthcare systems and infrastructure, and cultural and demographic differences.

In South Korea, many studies have used claims data to investigate the incidence of acute ischemic stroke. These studies assigned the ICD-10 diagnosis code I63 for hospitalized patients and used the results of imaging tests or drug claims to define the disease. The use of claims data allows the selection of a larger sample size and can provide insight into the incidence and treatment of acute ischemic stroke; however, the claims data have a limitation on the availability of clinical information, which can affect the accuracy of the research [12]. The use of claims data, such as ICD-10 diagnosis codes, imaging test codes, and medication codes, to identify acute ischemic stroke patients enable the identification of a higher number of patients compared with that using other methods. For example, a study published in 2013 that used these codes to identify patients with acute ischemic stroke in a particular country reported that the number of patients identified was twofold higher than that using a national registry database constructed by the Korean Stroke Society. This is because claims data can include information of patients who were not diagnosed with stroke by a neurologist or did not receive treatments for stroke but still had diagnosis codes in their medical records. It is difficult to identify actual patients diagnosed with acute ischemic stroke based on the claims data. Therefore, a new analytical tool using the latest ML technology is required. In our study, we aimed to apply the ML techniques to analyze the claims data in order to develop an appropriate algorithm for identifying acute ischemic stroke patients.

## Methods

### Study participants and the development cohort

We obtained the data from the Korean National Health Insurance Service (NHIS) database, which is linked to the National Health Insurance Service Ilsan Hospital (NHIMC) database. The NHIS covers compulsory health insurance for all citizens in South Korea and provides cost-free annual or biennial health screening examinations for all insured individuals. Since South Korea has a single-payer national health system, all medical records of covered inpatient and outpatient visits and the results of national health examinations are collected in the NHIS database, which includes diagnostic codes, procedures, prescriptions, medical costs, and personal information (e.g., age, sex, residential area, income level, and disability status). In this study, patients diagnosed with ischemic stroke were defined as those who were treated by a neurologist or identified through a review of the medical records of patients who visited Ilsan Hospital between 2015 and 2021. Suspected patients were defined as those who underwent at least one brain magnetic resonance imaging (MRI)/computed tomography (CT) scan, excluding those diagnosed with ischemic stroke. The control group consisted of patients with suspected and diagnosed ischemic stroke, matched by sex and age, and were selected at a 1:1 ratio. We used the NHIS–NSC data (NHIS-2022-1-757) from the NHIS. The authors declare that they have no conflict of interest with the NHIS. This study was approved by the Institutional Review Board of NHIS Ilsan Hospital (NHIMC-2022-01-001-001). All methods were performed in accordance with the Transparent Reporting of a Multivariable Prediction Model for Individual Prognosis or Diagnosis (TRIPOD) guidelines.

### Model development

In this study, we developed a prediction model for ischemic stroke. The model used 61 features including all available data from claims data: 1 rule-based operational definition, 5 personal information, 21 health examinations, 4 medical records, and 30 word-embedding variables. The embedding variables were based on the assumption that the codes frequently used in similar medical situations will have a higher probability of appearing, using a word-embedding technique to screen to a total of 2692 codes (633 diagnosis codes, 1841 procedure records, 100 procedure material codes, or 118 prescription records). The vector values of each code are determined based on their position relative to one another [13]. The total 2692 variables were reduced to 300 using term frequency and transformed into 30 embedding vectors (Additional file 1: Multimedia Appendix 1). Events were identified using the following definitions: patients diagnosed with ischemic stroke were defined as those who were treated by a neurologist or identified through a review of the medical records from 2015 to 2021.

**Fig. 1:**
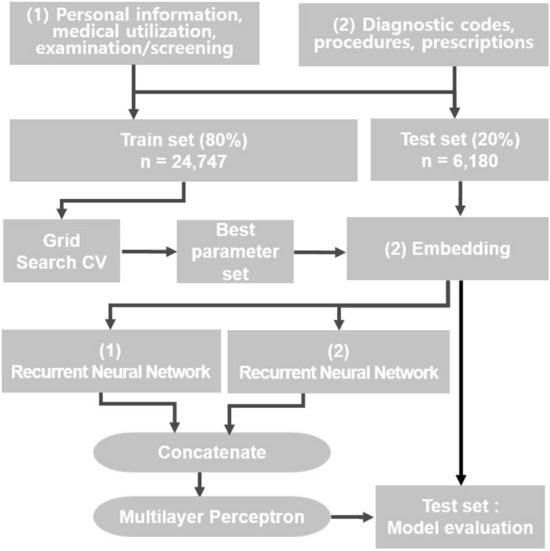
Overview of the model structure

We evaluated the performance of prediction models using multiple logistic regression, random forest, and extreme gradient boosting (XGBoost), which are tree-based ML techniques, and multi-layered perceptron, long–short-term memory (LSTM), gated recurrent unit (GRU), and convolutional neural network (CNN), which are neural network-based deep learning techniques. In neural network-based deep learning methods, an embedding model can be created using variables such as diagnosis, tests/treatments, and medication codes. A concatenated model, which combines the embedding model with additional variables such as qualifications, number of medical visits, and medical costs, can also be used for prediction (Fig. 1). In the medical usage variables, 2692 codes were used, including 633 disease codes, 1841 procedure codes, 100 treatment material codes, and 118 medication prescription codes. These codes were arranged in 402 variable values based on frequency of use and then padded to obtain the same number of variables by incorporating additional values, resulting in 300 variables. The statistical model or tree-based ML techniques used 300 variables in one-hot encoding, while the neural network-based deep learning methods used an embedding method to convert the variables into 30-dimensional vectors. The model features were summarized in 1-year intervals in 20 repetitions from 2002 to 2021. In the hyperparameter setting, Adam was used as the optimizer, the number of epochs was 100, the batch size was 64, the loss function used was binary cross-entropy, and early stopping was set using the Keras callback function (Additional file 2: Multimedia Appendix 2). These models used grid search to tune the hyperparameters.

The missing values were imputed using the last observation carried forward method, which replaces them with the previous data. The outliers that exceeded or were below the mean ± interquartile range were replaced with endpoint values. Standardization was performed by subtracting the minimum value from the original value and dividing it by the range (maximum value–minimum value). We utilized oversampling methods to tackle the data imbalance issue, irrespective of the prevalence of outcomes.

### Performance measurements

Model prediction validation was performed using AUROC, AUPRC, ROC curve, precision–recall curve, F_1_ score, precision, recall, precision–recall curve, accuracy, specificity, and calibration curve. The threshold value was defined as the point on the ROC curve where the sum of the estimated recall and specificity is maximized. The average of the product of recall and specificity was also used (Additional file 3: Multimedia Appendix 3).

### Validation

We performed the validation task in two ways. The data for model training and interval validation were divided into 80% and 20%. 80% of the model training data was used for model development, and 20% was used for model validation. All analyses were performed using Python (version 3.6.7) [14], and the model was built using the TensorFlow 1.14 [15] deep learning framework.

### Data availability

The data sets generated and/or analyzed in this study are not publicly available in accordance to the National Health Insurance Service regulations for the protection of electronic medical data.

## Results

### Demographic characteristics

We generated an ischemic stroke cohort based on the NHIS Ilsan Hospital data and established a patient group (3624 people), a disease-suspected group (15,902 people), and a normal control group (19,279 people) matched by sex and age at a ratio of 1:1. The data were combined with the National Health Information Database, and the mapping rate was 80%. The final cohort comprised 30,897 participants. A total of 1996 patients (10.09%) were included in the model training data, and the rule-based method used to analyze the training data of the patient group showed an accuracy of 91.36%. A total of 636 patients (10.38%) were included in the test data. The rule-based method used to analyze the training data of the patient group had an accuracy of 91.71% (Table 1).

**Table 1:** Demographic characteristics of the study cohorts

Characteristic	Development cohort		Validation cohort		Interval validation cohort	
	*n*	%	*n*	%	*n*	%
Patients(*n*, %)	19,773	100.00	4944	100.00	6180	100.00
Age(years, mean ± SD)	67.42 ± 20.19		67.58 ± 19.94		67.36 ± 20.02	
Gender(male, %)	9156	46.31	2318	46.89	2879	46.59
Income(*n*, %), medical aid	465	2.35	104	2.10	155	2.51
1	2054	10.39	543	10.98	638	10.32
2	2349	11.88	590	11.93	729	11.80
3	3748	18.96	942	19.05	1200	19.42
4	4398	22.24	1077	21.78	1389	22.48
5	6635	33.56	1668	33.74	2042	33.04
Death(*n*, %)	3731	18.87	919	18.59	1142	18.48
Rule-based method*(*n*, %)	18,065	91.36	4534	91.71	5663	91.63
Ischemic stroke (*n*, %)	1996	10.09	513	10.38	636	10.29

^*^The rule-based method is for individuals who are hospitalized with a diagnosis of I63 and have received anti-platelet therapy and anti-coagulant therapy within 30 days of diagnosis; *SD* standard deviation

### Model performance

**Fig. 2:**
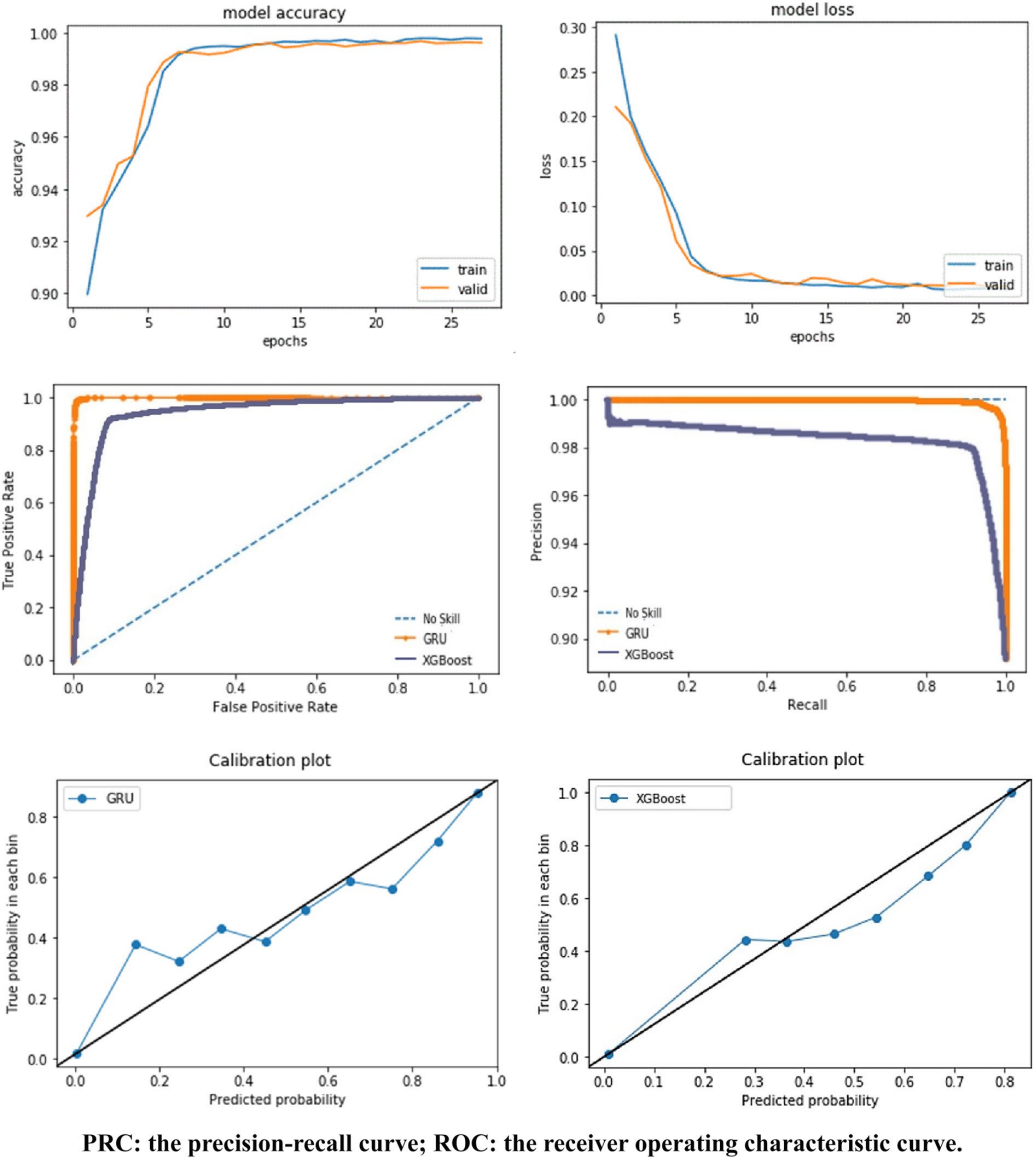
Results of accuracy, loss, ROC, PRC, and Calibration curve of these models

The rule-based method showed a high recall but relatively low precision in the test data (91.98% and 54.80%, respectively). The XGBoost model had a slightly low recall but showed high precision compared with the rule-based method (91.83% and 91.16%, respectively). The XGBoost method had relatively lower predictive performance compared to the neural network-based approach in terms of the F_1_ score (91.50%), the AUROC (94.46%), and the AUPRC (92.80%). The GRU model had a slightly higher accuracy rate of 99.81%. The F_1_ score was 99.81%, and the AUROC was the highest at 100.00% and the AUPRC was the highest at 100.00%. The deep learning model showed the highest prediction performance, and the one-dimensional CNN model demonstrated a fast convergence rate (epoch = 13). The accuracy and loss function used indicated the absence of problems, such as overfitting, in GRU. Using the Keras callback function, the early stopping and automatic saving of the training model were performed; the model converged when the epoch value was 27. When the prediction accuracy was compared between the XGBoost and GRU models based on the ROC curves and precision–recall curves, the GRU model was a slightly better fit than the XGBoost model. When the actual and predicted probabilities were compared through the calibration plots, the XGBoost model was underestimated, whereas the GRU model was fitted with no trend and was randomly distributed (Fig. 2).

### Feature importance

**Fig. 3:**
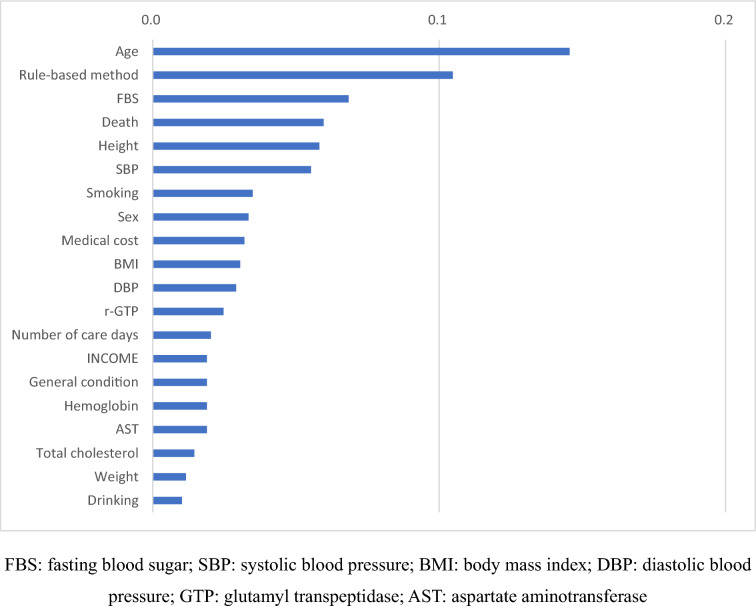
Feature importance: XGBoost model

The importance of model features was examined using the gain method, in which a decision is made based on the performance benefit obtained when a specific feature is divided. Age is the most important feature, followed by the rule-based method, fasting blood glucose level, death, height, highest blood pressure, smoking, sex, and so on. Income and total medical costs had a significant impact, and the medical utilization records, such as the number of days of care and hospitalization, were important variables that were explained (Fig. 3).

### Misclassification

**Fig. 4:**
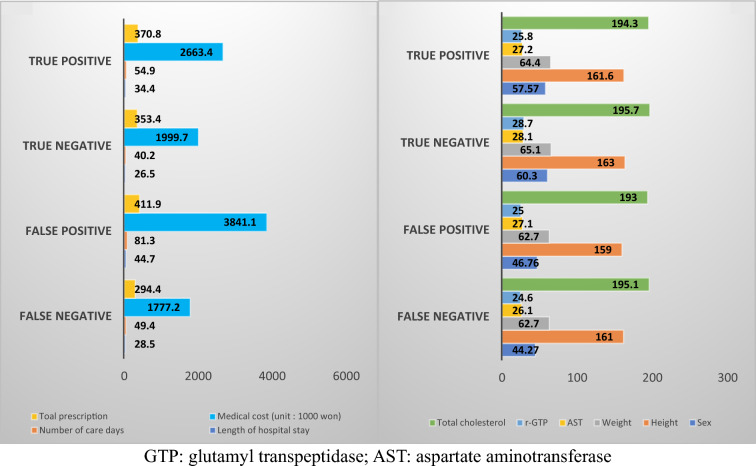
Characteristics of misclassified subjects based on the rule-based method

Upon examining the characteristics of the incorrect predicted group using the rule-based method, it was observed that the false positive group had higher healthcare expenses and utilization, while the true negative group showed relatively lower figures. Moreover, in the true negative group, notable elevations were observed in non-healthcare utilization factors such as height, weight, sex(male), aspartate aminotransferase, gamma-glutamyl transpeptidase, and total cholesterol levels (Fig. 4).

### Discussion

The rule-based method used for identifying ischemic stroke according to the claims data tends to classify more people as patients, resulting in a higher rate of false positives. In South Korea, where healthcare insurance is provided to all citizens and healthcare accessibility is high, this tendency is even more pronounced. In the past, when the medical insurance coverage included the cost for a brain MRI examination, the stroke incidence phenotyping by diagnostic code increased by 150% [16]. The rule-based method also predicted the stroke diagnosis with high recall but with relatively low precision. This tendency is a common phenomenon in hospital data, where individuals are classified as patients if they have any factor that raises suspicion for a disease [17]. In claims data, the likelihood of receiving insurance benefits is relatively high if an individual is diagnosed with a specific disease. We used ML to build a model with higher precision that can predict ischemic stroke with a recall capacity that is similar to that of the rule-based method. Our approach utilized a deep learning model on extensive healthcare billing data, deriving 30 word-embedding variables from frequently used codes. The prediction was made using a concatenated model that combined a deep learning model consisting of diagnostic, test, procedural, and prescription variables with another deep learning model comprising variables such as examinations, medical history, insurance level, mortality, and total medical expenses, apart from healthcare utilization variables. Among the ML models, the XGBoost model exhibited the highest accuracy. This model had a slightly lower recall but higher precision compared with the rule-based method, resulting in about 36% increase in its prediction accuracy. In addition, the AUROC and the AUPRC was highest in the XGBoost model. In this study, we also evaluated the advanced models such as RNNs and CNNs, which consider repeated measurements and time, in addition to the ML models. The predictive power was further improved in the deep learning model. The precision is improved compared to the rule-based model, and the recall is also improved. Among the deep learning models, GRU showed the highest accuracy (99.81%), precision (99.92%), recall (99.69%), AUROC (100.00%), and AUPRC (100.00%). This can be presented as an alternative to the operational definition of ischemic stroke (Table 2)

**Table 2:** AUROC, AUPRC, F_1_ score, precision, recall, accuracy, and specificity of each model

Operational definitions	AUROC	AUPRC	F_1_ score	Precision	Recall	Accuracy	Specificity
Rule-based method	91.47	73.39	68.68	54.80	91.98	91.47	91.41
Statistical models or tree-based machine learning techniques							
Logistic	90.76	88.90	86.39	84.45	88.43	86.06	83.68
Random Forest	92.70	91.70	79.03	92.55	68.96	81.68	94.43
XGBoost	94.46	92.80	91.50	91.16	91.83	91.45	91.08
Neural network-based deep learning techniques: with embedding							
MLP	98.00	98.00	94.82	95.41	94.24	94.85	95.45
LSTM	100.00	100.00	99.04	99.21	98.78	99.04	99.21
GRU	100.00	100.00	99.81	99.92	99.69	99.81	99.93
CNN	100.00	100.00	99.55	99.46	99.64	99.55	99.46

AUROC: the area under the receiver operating characteristic curve, AUPRC: the area under precision–recall curve, Rule-based method: individuals who are hospitalized with a diagnosis of I63 and have received anti-platelet therapy and anti-coagulant therapy within 30 days of diagnosis, *LSTM* long–short-term memory, *CNN* Convolutional Neural Networks, *MLP* Multi-Layer Perceptron, *GRU* Gated Recurrent Unit

To provide the gold standard definition, we used existing registry databases and collected data from patients who were treated directly by a neurologist or identified based on a chart review of mandatory records. However, this is not feasible in reality or incurs extremely high time and cost. Therefore, in previous studies, the rule-based method known as the silver standard has been widely used as an alternative to the gold standard. Our study showed that latest predictive models such as self-supervised learning, active learning, and semi-supervised learning models based on the silver standard can be considered as alternatives. For example, one could use a pretrained model to predict labels on a subset of randomly masked data, then compare the predicted labels to the actual labels, and finally fine-tune the model using the predicted label data. Notable examples include Bidirectional Encoder Representations from Transformers and generative pre-trained transformers (OpenAI), which can serve as alternative methods of performing the task. Therefore, the model can be enhanced using a combination of advanced models, such as transformer and CNN, rather than relying sole on traditional models. Because the variables were summarized in 1-year intervals in 20 repetitions, it was not possible to observed an improvement in the prediction performance of the latest models at arbitrary repetition points or when missing values occurred.

In previous studies that focused on claims data, the operational definition of the disease varied depending on the research purpose and expertise of researchers. According to the data surveyed in this study, when a disease was used as a feature, simple operational definitions using diagnostic codes alone were often employed. When used as a major label, the proportion of cases using operational definitions that incorporated additional imaging or medication codes in addition to the diagnostic codes was relatively high [17]. The proportion of studies using a definition that included imaging codes for ischemic stroke was 7.1%, which was higher compared with that of studies of other diseases, likely due to the fact that ischemic stroke can be easily diagnosed with imaging. In the previous studies, the diagnostic accuracy of the operational definition of ischemic stroke using ICD codes I60–I64 was approximately 43–64% [18–20]. Other previous studies that used the rule-based method of stroke included codes I64, I65, and I66 in addition to I63. This variability in phenotyping ischemic stroke is a major issue in studies that use claims data. The inclusion of additional codes, such as I65 or I66, in the analysis is less common, but the inclusion of the code I64, which is used when the cause of stroke is unclear, is more common [17]. Including the I64 code in the analysis may result in a slightly decreased positive predictive value for ischemic stroke, but it allows a larger number of patients with ischemic stroke to be included in the study [21]. A previous study that included I63 and I64 codes for the diagnosis of ischemic stroke and used additional criteria such as hospitalization and medication codes for validation found that despite the increased sensitivity achieved by including I64, the specificity remained low (< 50%) [22].

This study is the first to use claims data to construct an ML model and a deep learning model that can accurately identify ischemic stroke, thus surpassing the accuracy of previous studies. Since the ML technique greatly improved precision compared to the rule-based method, it will be possible to increase the accuracy of research results using ischemic stroke as an outcome variable. Using claims data, the study was able to obtain a large amount of patient information, enabling model validation on a wide variety of cases. The use of advanced ML and deep learning techniques also allows the model to identify complex patterns and relationships in the data, leading to improved diagnostic accuracy. Additionally, the recurrent neural network approach in deep learning was utilized, given its ability to effectively handle the characteristic irregular and repetitive nature of nationwide extensive healthcare claim data, showcasing its applicability. In studies using claims data, the results obtained using traditional rule-based methods of ischemic stroke should be compared with those obtained using the model developed in this study. Upon examining the characteristics of the incorrect predicted group using the rule-based method, the false positive group had higher healthcare costs, while the true negative group had lower figures, accurately reflecting the billing data nature. Such an analysis can then be used to make adjustments to the model, such as incorporating additional variables or using different ML techniques, to improve the diagnostic accuracy. As an increasing number of studies utilize prediction models, the reliability of research results related to ischemic stroke based on claims data may increase.

However, this study has some limitations. First, variables related to healthcare utilization may be affected by policies and medical insurance fees applied at the time, as well as advances in medical technology; therefore, the aspect of being dependent on the era should be taken into consideration when developing a model. This is not just a problem for operational definition prediction models; rather, it must be sufficiently reflected in the study. Therefore, it is necessary to review the generalizability of the models by splitting the data in chronological order or regularly updating the prediction models. Second, the model was based on the data obtained from a single institution in a particular country. The clinical characteristics of patients with stroke may vary depending on the medical institution, and the same model may be difficult to data from other countries with different medical insurance systems. To complement this, studies should be conducted using databases [23] of various South Korean institutions in the future.

## Conclusion

This study found that ML prediction models can improve the predictability of operational definitions that have relied on rule-based methods employed in previous studies using claims data. This can provide processed and refined disease variables rather than primitive data, such as diagnosis codes, calculation special codes, medication, test and procedure codes, examination items, and qualification information when conducting studies based on claims data. Therefore, it can quickly and accurately derive the study results when conducting future studies using big data.

### Supplementary Information

**Additional file 1: **The input features of the model.

**Additional file 2: **Logistic regression, Random Forest, XGBoost, LSTM, and GRU hyperparameters.

**Additional file 3: **Threshold values of each models.

## Data Availability

Not applicable.
