# Supplementary material for: Use of machine learning techniques for identifying ischemic stroke instead of the rule-based methods: a nationwide population-based study
Source: Eur J Med Res. 2024 Jan 3;29:6. doi: 10.1186/s40001-023-01594-6 (PMC10763197; doi:10.1186/s40001-023-01594-6)
Supplement: Supplementary file 1 — Additional file 1: The input features of the model. [file 40001_2023_1594_MOESM1_ESM.docx]

**Appendix 1.** **The input features of the model**

| **Category** | **Input features** |
| --- | --- |
| Rule-based method(1) | Rule-based method |
| Personal informations(5) | Age, sex, income, disability, death |
| Health examinations(21) | Smoking, exercise, level of exercise, drink, level of drink, BMI, diastolic blood pressure, systolic blood pressure, fasting blood sugar, r-GTP, current status, drinking needs improvement, exercise needs improvement, smoking needs improvement, weight needs improvement, hemoglobin, height, weight, AST(SGOT), ALT(SGPT), total cholesterol, trauma and fatigue, urine protein |
| Medical records(4) | Hospitalization days, care days, total prescriptions, medical costs |
| Diagnosis codes(633) | Cholera(A00), Typhoid and paratyphoid fevers(A01), ..., Liver transplant(Z944) |
| Procedure records(1841) | Classified by the third code of the classification number of procedures of the Health Insurance Review and Assessment Service |
| Procedure material codes(100) | Classified by the classification number of procedure materials of the Health Insurance Review and Assessment Service |
| Prescriptions records(118) | Classified by the classification number of prescriptions of the Health Insurance Review and Assessment Service |
