# Supplementary material for: Use of machine learning techniques for identifying ischemic stroke instead of the rule-based methods: a nationwide population-based study
Source: Eur J Med Res. 2024 Jan 3;29:6. doi: 10.1186/s40001-023-01594-6 (PMC10763197; doi:10.1186/s40001-023-01594-6)
Supplement: Supplementary file 2 — Additional file 2: Logistic regression, Random Forest, XGBoost, LSTM, and GRU hyperparameters. [file 40001_2023_1594_MOESM2_ESM.docx]

**Appendix 2. Logistic regression, Random Forest, XGBoost, LSTM, and GRU hyperparameters**

Logistic regression Hyperparameters

| Penalty | l2 |
| --- | --- |
| Optimization | lbfgs |
| Max Iteration | 100 |
| Regularization strength | 1.0 |

Random Forest hyperparameters

| Bootstrap | True |
| --- | --- |
| Max depth | None |
| Max features | Auto |
| Min samples leaf | 1 |
| Min samples split | 2 |
| N estimators | 10 |
| Criterion | Gini |

XGBoost hyperparameters – Grid search

| Max depth | 3 |
| --- | --- |
| Learning rate | 0.03 |
| Gamma | 0 |
| Booster | gbtree |
| Min child Weight | 1 |
| Objective | Binary logistic regression |

LSTM or GRU hyperparameters

| Optimizer | Adam |
| --- | --- |
| Learning rate | 0.05 |
| Batch size | 64 |
| Maximum epochs | 100 |
