# Supplementary material for: Use of machine learning techniques for identifying ischemic stroke instead of the rule-based methods: a nationwide population-based study
Source: Eur J Med Res. 2024 Jan 3;29:6. doi: 10.1186/s40001-023-01594-6 (PMC10763197; doi:10.1186/s40001-023-01594-6)
Supplement: Supplementary file 3 — Additional file 3: Threshold values of each models. [file 40001_2023_1594_MOESM3_ESM.docx]

**Appendix 3. Threshold values of each models**

| **Models** | **Threshold values** |
| --- | --- |
| Statistical models or tree-based machine learning techniques | |
| Logistic | 0.5163 |
| Random Forest | 0.4000 |
| XGBoost | 0.2800 |
| Recurrent neural network based deep learning techniques | |
| MLP | 0.9580 |
| LSTM | 0.8273 |
| GRU | 0.7527 |
| CNN | 0.7058 |

The threshold value is determined as the point on the ROC curve where the sum of the estimated recall and specificity is maximized. It was also considered to use the average of the product of recall and specificity.
